# Elevated stress hyperglycemia and the presence of intracranial artery stenosis increase the risk of recurrent stroke

**DOI:** 10.3389/fendo.2022.954916

**Published:** 2023-01-09

**Authors:** Yongle Wang, Hongxuan Fan, Weiying Duan, Zhaoyu Ren, Xuchang Liu, Tingting Liu, Yanan Li, Kaili Zhang, Haimei Fan, Jing Ren, Juan Li, Xinyi Li, Xuemei Wu, Xiaoyuan Niu

**Affiliations:** ^1^ Department of Neurology, First Hospital of Shanxi Medical University, Taiyuan, Shanxi, China; ^2^ Clinical College, Shanxi Medical University, Taiyuan, Shanxi, China; ^3^ Department of Cardiology, Second Hospital of Shanxi Medical University, Taiyuan, Shanxi, China; ^4^ Department of Neurology, The Bethune Hospital of Shanxi Province, Taiyuan, Shanxi, China; ^5^ Department of Neurology, Sixth Hospital of Shanxi Medical University (General Hospital of Tisco), Taiyuan, Shanxi, China

**Keywords:** stress hyperglycemia, intracranial atherosclerotic stenosis, ischemic stroke, stroke recurrence, fasting blood glucose

## Abstract

**Background:**

Stress hyperglycemia has served as a reliable biomarker to predict poor outcomes after ischemic stroke. However, recent studies have reported some contrary conclusions. Different stroke subtypes may respond inconsistently to stress hyperglycemia. The progression of intracranial atherosclerotic stenosis (ICAS) is tightly related to hyperglycemia. Thus, this study aims to determine the relationship between stress hyperglycemia and recurrent stroke in ischemic stroke patients with or without intracranial atherosclerotic stenosis.

**Methods:**

This is a multicenter retrospective observational cohort study. Patients with acute minor ischemic stroke and eligible computed tomography and magnetic resonance imaging data were enrolled. The severity of stress hyperglycemia is measured by the stress hyperglycemia ratio (SHR). SHR was calculated based on fasting plasma glucose (FPG) and hemoglobin A1c (HbA1c) levels. The primary outcome was stroke recurrence during hospitalization. The interaction of SHR levels with the presence of ICAS on the primary outcome was investigated using univariable and multivariable Cox proportional hazards models. Restricted cubic splines were applied to determine the nonlinear relationship between SHR and primary outcome. A two-piecewise linear regression model was used to identify the threshold of SHR.

**Results:**

A total of 610 participants were included in the study. The average age of the patients was 61.4 ± 12.9 years old, and approximately 70% of participants were males. A total of 189 (30.98%) patients had ICAS. The patients were categorized into 3 groups based on the tertiles of SHR. Compared with the group with a lower SHR, a higher SHR was significantly associated with the risk of stroke recurrence in the ICAS group (hazard ratio [HR], 8.52, 95% confidence interval [CI], 3.16-22.96, P<0.001). When SHR was treated as a continuous variable, each 0.1-unit increase in SHR in the ICAS group was associated with a 1.63-fold increase in the risk of recurrence (HR, 1.63, 95% CI, 1.39-1.9, P<0.001) with a threshold of 0.75. FPG but not HbA1c was associated with stroke recurrence in ICAS patients (HR, 1.17, 95% CI, 1.08-1.26, P<0.001). Sensitive analyses showed consistent results after adjusting for previous diabetes mellitus, oral hypoglycemic agents and insulin injection.

**Conclusions:**

SHR represents a better biomarker to predict the risk of stroke recurrence in patients with ICAS than FPG and HbA1c regardless of previous diabetes mellitus.

**Trial registration:**

https://www.chictr.org.cn/showproj.aspx?proj=125817; Identifier, [ChiCTR2100046958].

## 1 Introduction

Ischemic stroke is a leading cause of death and long-term disability worldwide (1), and the economic costs of treatment and poststroke care are substantial. Intracranial atherosclerotic stenosis (ICAS) of a major intracranial artery is one of the most common causes of minor stroke worldwide and is more prevalent in Asian, black and Hispanic patients. In contrast, extracranial carotid atherosclerotic stenosis (ECAS) is more common in white patients (2). ICAS is associated with a high risk of recurrent stroke compared with other stroke subtypes (3) because arterial stenosis makes blood flow more turbulent and can induce rupture of atherosclerotic plaques or thrombus formation, causing subsequent embolism (4). The Chinese Intracranial Atherosclerosis (CICAS) study showed that ICAS is present in 46.6% of stroke patients in China and confirmed that ICAS is an independent risk factor for stroke recurrence (2). Secondary analysis of two large randomized controlled trials, Warfarin–Aspirin Symptomatic Intracranial Disease (WASID) and the Stenting and Aggressive Medical Management for Preventing Recurrent stroke in Intracranial Stenosis (SAMMPRIS) study, illustrated that symptomatic intracranial stenosis > 70% significantly increased the risk of stroke recurrence (5, 6). This conclusion has also been confirmed in minor stroke or transient ischemic attack (TIA) patients characterized by a higher rate of recurrence and early neurologic deterioration during hospitalization (7).

Previous studies have shown that stress hyperglycemia predicts increased risks of in-hospital mortality and poor functional recovery after ischemic stroke (8, 9). Stress hyperglycemia is defined as hyperglycemia occurring in the acute phase of critical disease, such as stroke, myocardial infarction and hemorrhagic shock. Moreover, stress hyperglycemia occurs regardless of preexisting diabetes mellitus. In contrast to chronic hyperglycemia, stress hyperglycemia reflects the inflammatory and neurohormonal dysregulations that occur during a major illness and is strongly associated with adverse outcomes (10). The direct impact of stress hyperglycemia on endothelial cells, which is characterized by increased the activation of prothrombotic factors, such as fibrinopeptide A and factor VII, and decreased plasma fibrinolytic activity, represents a plausible mechanism (11–13). Moreover, evidence that lowering glucose with insulin reduces ischemic brain damage in animal models of stroke suggests that stress-induced hyperglycemia may be a risk factor for brain damage (8). Previous studies have shown that chronic hyperglycemia is one of the most important contributors to ICAS (14). Hyperglycemia arises in 30–40% of people with acute ischemic stroke (15). In the acute phase of ischemic stroke caused by ICAS, the narrowed vessel on the infarcted side is more vulnerable to hemodynamic fluctuation and elevated blood viscosity induced by stress hyperglycemia, which may lead to an increased risk of stroke recurrence. There have been several parameters, such as fasting glucose, glycemic gap and stress hyperglycemia ratio (SHR) to measure the extent of stress hyperglycemia, among which SHR provided the best prognostic insight at admission to assess the relationship between stress hyperglycemia and ischemic stroke outcome (16). SHR was calculated based on hemoglobin A1c (HbA1c) and fasting plasma glucose (FPG). HbA1c was used to estimate the average blood glucose before stroke onset, based on the equation derived by Nathan et al. as follows: Estimated average glucose = [1.59 * HbA1c (%)] – 2.59. SHR was then calculated as FPG divided by the estimated average glucose. Several clinical studies have confirmed the relationship between stress hyperglycemia and stroke recurrence, especially for minor ischemic stroke patients (The National Institutes of Health Stroke Scale [NIHSS] ≤5) (17, 18). However, the interaction or combined effects of stress hyperglycemia and ICAS on patients with acute minor stroke remain uncertain.

Thus, in this study, we assessed the association between stress hyperglycemia level and recurrence risk in acute ischemic minor stroke patients with or without ICAS.

## 2 Materials and methods

### 2.1 Study design

This study is a multicenter retrospective observational cohort study based on real-world research conducted at three advanced stroke centers in Shanxi Province (First Hospital of Shanxi Medical University, Bethune Hospital of Shanxi Province, Sixth Hospital of Shanxi Medical University (General Hospital of Tisco). The study included patients with acute minor ischemic stroke (NIHSS score ≤ 5) treated with antiplatelet therapy within 72 h of symptom onset. Diagnosis of the index stroke was confirmed by the attending neurologist according to the World Health Organization definition and was based on medical history, clinical presentation, and findings in neuroimaging (computed tomography or magnetic resonance imaging). We retrieved the electronic medical records of all patients hospitalized for minor ischemic stroke during four months each year (March, June, September, December) from 2010 to 2018.

### 2.2 Inclusion and exclusion criteria

The inclusion criteria were as follows: (1) patients with ischemic stroke within 72 hours of onset; (2) NIHSS score ≤ 5 at onset or NIHSS score > 5 at onset, but symptoms were relieved before antiplatelet therapy; (3) three-dimensional time-of-flight magnetic resonance angiography (MRA) or computed tomography angiography (CTA) was performed on admission. The exclusion criteria were as follows: (1) modified Rankin score (MRS) > 2, (2) TIA, (3) recombinant tissue plasminogen activator (rt-PA) treatment, (4) mechanical thrombectomy or stent-assisted angioplasty, (5) participation in other clinical trials, and (6) cardiogenic stroke. Cardiogenic stroke is defined according to the Trial of Org 10172 in Acute Stroke Treatment (TOAST) criteria as arterial occlusions presumably due to single or multiple emboli from the heart. At least one cardiac source for the occlusion, such as atrial fibrillation, mechanical prosthetic valve or left ventricular thrombus, must be identified for the diagnosis of cardioembolic stroke. Potential large-artery atherosclerotic sources and any other causes of stroke do not support the above diagnosis (19). Patients who are diagnosed with bleeding or other pathological diseases, such as vascular malformations, tumors, abscesses or other major nonischemic brain diseases (such as multiple sclerosis), on baseline head computed tomography (CT) or magnetic resonance imaging (MRI) are not eligible for the study. Glucose and glycosylated hemoglobin measurements that were not recorded in the hospital were also excluded.

### 2.3 Baseline data collection

Baseline data included demographics, stroke features, and vascular risk factors. All the data were directly obtained from a prespecified anonymous case report form (CRF) completed by trained physicians and nurses after searching every hospital’s electronic medical record system. Demographics included age, sex, body mass index, systolic pressure, and diastolic pressure. Stroke features included hours from symptom onset to admission, stroke subtype, classified according to TOAST criteria, and baseline NIHSS score. Vascular risk factors, such as hypertension (HTN), history of stroke, TIA, myocardial infarction (MI), coronary atherosclerotic heart disease (CHD), peripheral arterial disease (PAD), diabetes mellitus, hyperlipidemia, history of cerebral hemorrhage and smoking, were also collected. Smoking was defined as current smokers who had smoked more than 100 cigarettes in their lifetime and had smoked in the 30 days preceding the stroke. Laboratory findings, including homocysteine (HCY), high-density lipoprotein cholesterol (HDL-C), low-density lipoprotein cholesterol (LDL-C), triglycerides (TGs), total cholesterol (TC), hemoglobin A1c (HbA1c), and fasting plasma glucose (FPG), were collected. Medication information included data on antiplatelet medication at admission, statin therapy, insulin injection and oral hypoglycemic agents. All patients received antiplatelet and statin medication within 72 hours of stroke onset.

### 2.4 Assessment of stress hyperglycemia

All patients had venous blood drawn within the first 24 hours after admission. FPG and HbA1c were then measured. HbA1c was used to estimate the average blood glucose prior to admission based on the equation derived by Nathan et al. as follows: Estimated average glucose = [1.59 * HbA1c (%)] – 2.59 (20). SHR was then calculated as FPG (mmol/L) divided by the estimated average glucose. The patients were then categorized into 3 groups based on tertiles of SHR (low, middle and high) for further comparisons.

### 2.5 Imaging collection and analysis

All eligible CT and MRI scans of individual patients were collected from centers participating in the imaging subgroup analysis. For MRI, T1-weighted imaging, T2-weighted imaging, diffusion-weighted imaging, and 3D time-of-flight MRA were needed for the analysis. ICAS was determined by the presence of 50%–99% stenosis according to Warfarin-Aspirin Symptomatic Intracranial Disease (WASID) trial criteria or occlusion of at least one of the following arterial segments: intracranial internal carotid artery, A1 or A2 of anterior cerebral artery, M1 or M2 of middle cerebral artery, P1 or P2 of posterior cerebral artery, posterior inferior cerebellar artery, vertebral artery, and basilar artery (21). If MRI is not available, CT angiography can be used instead to assess the degree of cerebral vascular stenosis. For any potential ICAS lesion, disagreements regarding the degree of stenosis were resolved by consulting with a third experienced physician. We retrospectively collected all data described above from the hospital’s electronic medical record system. The study subjects were divided into two groups according to the presence of ICAS.

### 2.6 Study outcome

The primary outcome was stroke recurrence, including new ischemic stroke, hemorrhagic stroke, and TIA, during hospitalization. Due to the long inclusion span, most patients lack follow-up information after discharge. Thus, this paper only studied the in-hospital recurrence. Recurrence of ischemic stroke was defined as acute focal cerebral or retinal infarction as follows: an increase in the NIHSS score of four or more, new infarction or enlargement of the original focus on MRI or CT. Hemorrhagic stroke was defined as acute infiltration of blood into the brain parenchyma or subarachnoid space with associated neurological symptoms and imaging findings (22). TIA was defined as a transient episode of neurological dysfunction without acute cerebral infarction, as described previously (23).

### 2.7 Statistical analysis

Categorical variables are presented as frequencies and percentages, and continuous variables are presented as the means (standard deviations) or medians (interquartile ranges). Intergroup differences for categorical variables were analyzed using Pearson’s chi-squared test or Fisher’s exact test, and continuous variables were analyzed using Student’s t test, Analysis of Variance (ANOVA), Kruskal−Wallis test or the Wilcoxon rank-sum test, as appropriate. For the chi-squared test, at least 80% of the cells in the contingency table must have more than 5 counts. Otherwise, the test is unreliable, and Fisher’s exact test was employed. Between the included and excluded groups, Student’s t test was applied when the distributions of the two independent samples were normal; otherwise, the Wilcoxon rank-sum test was used. To compare continuous variables among different tertiles of SHR, ANOVA (parametric) or the Kruskal−Wallis (nonparametric) test were used. The interaction of SHR levels with ICAS on the study outcome was investigated using crude and multivariable Cox proportional hazards models. Confounders were identified based on univariable analysis and previous reports. Model I was adjusted for age and sex. Model II was adjusted for age, sex, smoking, NIHSS, stroke history, antiplatelet therapy. Model III was adjusted for age, sex, systolic pressure, diastolic pressure, smoking, body mass index (BMI), NIHSS, history of stroke, hyperlipoidemia, antiplatelet medication at admission and statin therapy. Models IV and V further adjusted for diabetes mellitus and hypoglycemic treatment as sensitivity analyses. Subgroup and interaction analyses according to history of diabetes mellitus were performed. The nonlinear relationship between SHR levels and recurrent stroke was assessed by restricted cubic splines, and a two-piecewise linear regression model was applied to identify the threshold of the smoothing curve. All tests were two-sided, and a P value of 0.05 was considered to indicate statistical significance. A Kaplan−Meier curve was drawn and compared by the log-rank test. To maximize statistical power and minimize bias that might occur, we used multiple imputation with chained equations to impute missing values. We repeated all analyses with the complete data cohort for comparison. The results were consistent with the present analysis. A post hoc power analysis was performed to see the sampling variation, by simulating the power of set parameters of regression and interaction analysis. Statistical analysis was performed using the statistical software package R (http://www.R-project.org, The R Foundation).

## 3 Results

### 3.1 Clinical characteristics among SHR groups

**Figure 1:**
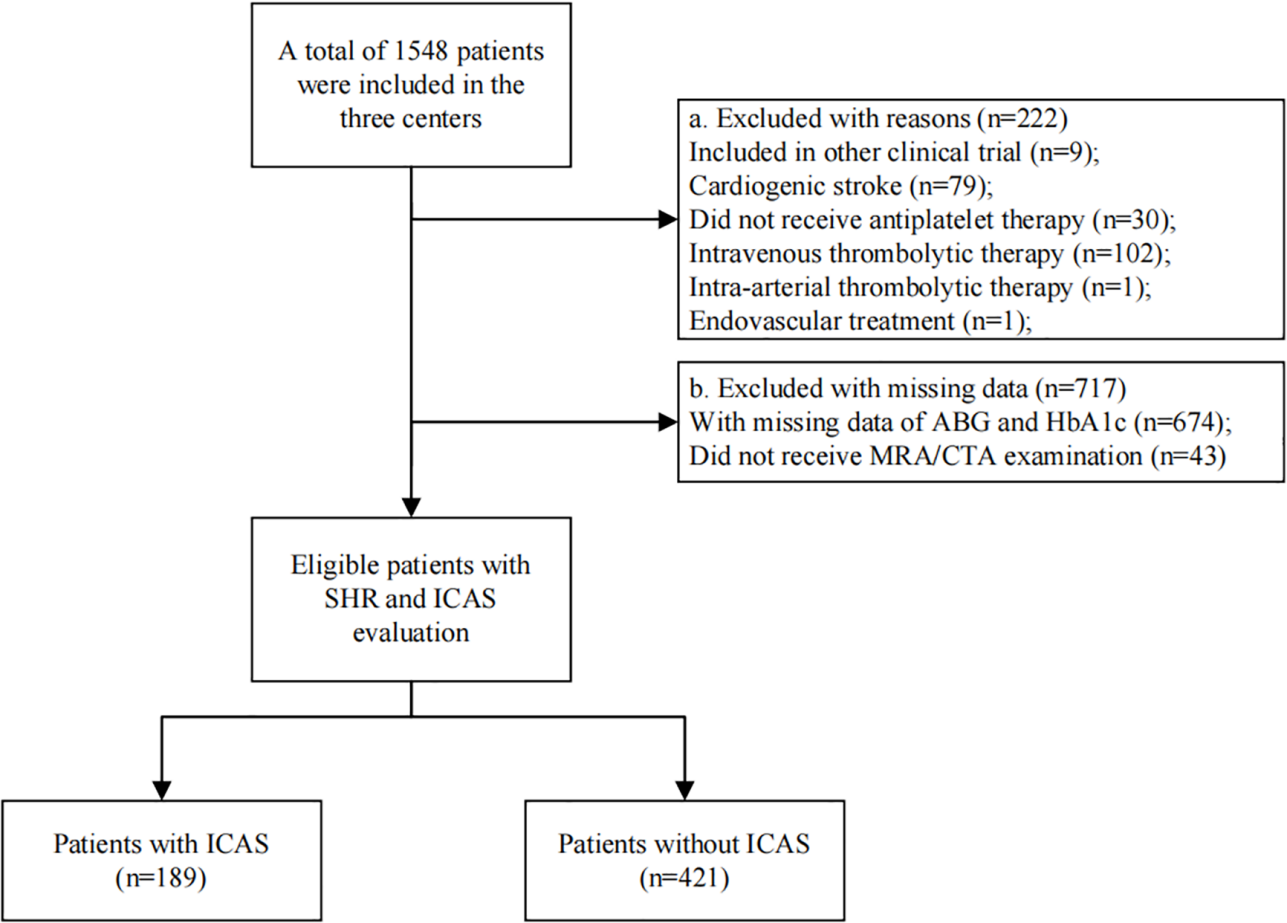
Flowchart of patient selection. There were 610 eligible patients included in the final analysis. Patients were grouped according to whether they have ICAS or not.

A total of 610 patients were included in the study, and the specific flowchart is shown in **Figure 1**. The clinical characteristics of the patients included and those excluded were well balanced except for a higher proportion of smoking, diabetes mellitus, and statin use in the included group (**Table 1**). The baseline characteristics of all the participants are shown in **Table 2**. The mean age was 61.4 ± 12.9 years, and approximately 70% were male. The study included 189 (30.89%) patients with ICAS. Patients with higher SHR were more likely to have previous diabetes mellitus, higher FPG, and lower HCY but less likely to have hyperlipoidemia. No significant difference in the incidence of ICAS was noted among the different SHR groups. During in-hospital follow-up, a total of 58 (9.5%) patients experienced stroke recurrence. Patients in the higher SHR group endured more recurrence than those in the lower SHR group.

**Table 1:** Baseline characteristics of patients included and excluded.

Variables	Total (n = 1494)	Excluded (n = 884)	Included (n = 610)	*p*-Value
Female, n (%)	436 (29.2)	250 (28.3)	186 (30.5)	0.386
Age, Median (IQR)	62.0 (53.0, 72.0)	62.0 (53.0, 73.0)	61.0 (53.0, 71.0)	0.134
BMI, Median (IQR)	24.9 ± 3.1	24.8 ± 3.4	24.9 ± 4.0	0.697
Smoking, n (%)	644 (43.1)	349 (39.5)	295 (48.4)	< 0.001
Hypertension, n (%)	880 (58.9)	522 (59)	358 (58.7)	0.931
Diabetes, n (%)	316 (21.2)	160 (18.1)	156 (25.6)	< 0.001
Hyperlipoidemia, n (%)	64 (4.3)	31 (3.5)	33 (5.4)	0.098
AF history, n (%)	51 (3.4)	34 (3.8)	17 (2.8)	0.335
Stroke history, n (%)	348 (23.3)	201 (22.7)	147 (24.1)	0.583
Cerebral hemorrhage, n (%)	23 (1.5)	14 (1.6)	9 (1.5)	1
CHD, n (%)	90 (6.0)	46 (5.2)	44 (7.2)	0.135
AMI, n (%)	44 (2.9)	22 (2.5)	22 (3.6)	0.271
PAD, n (%)	3 (0.2)	1 (0.1)	2 (0.3)	0.571
Antiplatelet therapy, n (%)				0.592
Aspirin	564 (37.8)	339 (38.3)	225 (36.9)	
DAPT	809 (54.1)	452 (51.1)	357 (58.5)	
Clopidogrel	121 (8.1)	93 (10.5)	28 (4.6)	
Statins therapy, n (%)	532 (35.6)	275 (31.1)	257 (42.1)	< 0.001
Oral hypoglycemic agents	110 (7.4)	67 (7.6)	43 (7.0)	0.481
Insulin injection	59 (3.9)	37(4.2)	22 (3.6)	0.298
NIHSS, Median (IQR)	2.0 (1.0, 3.0)	2.0 (1.0, 3.0)	2.0 (1.0, 3.0)	0.101

BMI, body mass index; AF, atrial fibrillation; CHD, coronary atherosclerotic heart disease; MI, myocardial infarction; PAD, peripheral arterial disease; DAPT, dual antiplatelet therapy; NIHSS, the National Institutes of Health Stroke Scale. Categorical variables are presented as frequencies and percentages, and continuous variables are presented as the means (standard deviations) or medians (interquartile ranges). Intergroup differences of categorical variables were analyzed using Pearson’s chi-squared test or Fisher’s exact test, and continuous variables were analyzed using Student’s t test or the Wilcoxon rank-sum test, as appropriate. A P value of 0.05 is considered to indicate statistical significance.

**Table 2:** Clinical characteristics among tertiles of SHR.

Variables	Total (n = 610)	Lower SHR (n = 203)	Middle SHR (n = 203)	Higher SHR (n = 204)	*p*-Value
Demographics					
Female, n (%)	186 (30.5)	63 (31)	56 (27.6)	67 (32.8)	0.504
Age, year, Mean ± SD	61.4 ± 12.9	62.8 ± 12.5	60.3 ± 13.1	61.1 ± 13.1	0.143
BMI, Mean ± SD	24.7 ± 3.5	24.6 ± 3.9	24.9 ± 3.4	24.6 ± 3.1	0.639
Smoking, n (%)	295 (48.4)	91 (44.8)	99 (48.8)	105 (51.5)	0.403
Systolic, mmHg, Mean ± SD	146.5 ± 21.2	144.8 ± 20.3	146.6 ± 21.4	148.2 ± 21.7	0.258
Diastolic, mmHg, Mean ± SD	87.9 ± 13.6	88.1 ± 13.1	88.0 ± 14.2	87.5 ± 13.4	0.92
Medical History					
Hypertension, n (%)	358 (58.7)	113 (55.7)	121 (59.6)	124 (60.8)	0.547
Diabetes, n (%)	156 (25.6)	39 (19.2)	48 (23.6)	69 (33.8)	0.002
Hyperlipoidemia, n (%)	33 (5.4)	18 (8.9)	12 (5.9)	3 (1.5)	0.004
AF, n (%)	12 (2.0)	5 (2.5)	3 (1.5)	4 (2)	0.826
Stroke, n (%)	152 (24.9)	48 (23.6)	50 (24.6)	54 (26.5)	0.799
TIA, n (%)	18 (3.0)	8 (3.9)	5 (2.5)	5 (2.5)	0.594
Cerebral hemorrhage, n (%)	9 (1.5)	3 (1.5)	3 (1.5)	3 (1.5)	1
CHD, n (%)	45 (7.4)	17 (8.4)	9 (4.4)	19 (9.3)	0.136
AMI, n (%)	22 (3.6)	6 (3)	8 (3.9)	8 (3.9)	0.831
PAD, n (%)	4 (0.7)	3 (1.5)	1 (0.5)	0 (0)	0.139
Treatment at admission					
Antiplatelet therapy, n (%)					0.247
Aspirin	225 (36.9)	67 (33)	81 (39.9)	77 (37.7)	
Aspirin plus Clopidogrel	357 (58.5)	122 (60.1)	114 (56.2)	121 (59.3)	
Clopidogrel	28 (4.6)	14 (6.9)	8 (3.9)	6 (2.9)	
Statins therapy, n (%)	257 (42.1)	87 (42.9)	81 (39.9)	89 (43.6)	0.724
Oral hypoglycemic agents	43 (7.0)	10 (4.9)	12 (5.9)	21 (10.3)	0.079
Insulin injection	22 (3.6)	3 (1.5)	7 (3.4)	12 (5.9)	0.058
NIHSS, Median (IQR)	2.0 (1.0, 4.0)	2.0 (1.0, 4.0)	2.0 (1.0, 4.0)	2.0 (1.0, 3.0)	0.805
Hours from onset, hour, Median (IQR)	26.0 (11.0, 48.0)	28.0 (12.0, 48.0)	28.0 (16.0, 48.0)	21.0 (10.0, 48.0)	0.078
HDL, mmol/L, Mean ± SD	1.0 ± 0.3	1.0 ± 0.3	1.0 ± 0.3	1.0 ± 0.3	0.338
LDL, mmol/L, Mean ± SD	2.4 ± 0.8	2.3 ± 0.8	2.4 ± 0.8	2.5 ± 0.8	0.108
TG, mmol/L, Mean ± SD	1.6 ± 1.0	1.5 ± 0.9	1.6 ± 1.0	1.8 ± 1.1	0.068
TC, mmol/L, Mean ± SD	4.1 ± 1.1	4.1 ± 1.0	4.2 ± 1.1	4.2 ± 1.1	0.463
HCY, μmol/L Median (IQR)	17.4 (13.3, 24.3)	17.8 (14.5, 24.0)	18.7 (13.3, 26.3)	16.1 (11.9, 21.9)	0.002
HbA1c, %, Mean ± SD	6.2 ± 1.6	6.4 ± 1.4	6.2 ± 1.5	6.1 ± 1.8	0.06
FPG, mmol/L, Median (IQR)	5.6 (4.9, 7.2)	4.9 (4.4, 5.5)	5.6 (5.0, 6.8)	6.9 (5.8, 9.2)	< 0.001
SHR, Mean ± SD	0.9 ± 0.2	0.7 ± 0.1	0.9 ± 0.1	1.2 ± 0.2	< 0.001
ICAS, n (%)	189 (31.0)	66 (32.5)	60 (29.6)	63 (30.9)	0.812
Length of hospitalization, day, Mean ± SD	11.5 ± 5.1	11.7 ± 5.3	12.0 ± 4.8	10.9 ± 5.1	0.081
Incidence of recurrence, n (%)	58 (9.5)	10 (4.9)	12 (5.9)	36 (17.6)	< 0.001

LDL-C, low-density lipoprotein cholesterol; HDL-C, high-density lipoprotein cholesterol; TG, triglycerides; TC, total cholesterol; HCY, homocysteine; FPG, fasting plasma glucose; SHR, stress hyperglycemia ratio. Categorical variables are presented as frequencies and percentages, and continuous variables are presented as the means (standard deviations) or medians (interquartile ranges). Intergroup differences of categorical variables were analyzed using Pearson’s chi-squared test or Fisher’s exact test, and continuous variables were analyzed using ANOVA or the Kruskal−Wallis test, as appropriate. A P value of 0.05 was considered to indicate statistical significance.

### 3.2 Univariate and multivariate analyses of factors associated with stroke recurrence

Univariate and multivariate Cox regression analyses were performed to determine factors associated with stroke recurrence. Univariate Cox regression analysis is shown in **Table 3**. Diabetes mellitus (HR, 1.97; 95% CI, 1.16-3.34, P=0.011), stroke history (HR, 1.75; 95% CI, 1.03-2.99, P=0.04), higher SHR (HR, 3.85; 95% CI, 1.91-7.75, P<0.001), and ICAS (HR, 9.41; 95% CI, 4.98-17.77, P<0.001) were associated with in-hospital stroke recurrence (P<0.05). The univariate results tentatively suggested that ICAS and SHR levels were associated with an increased risk of in-hospital stroke recurrence.

**Table 3:** Univariate analyses of factors associated with stroke recurrence.

Univariates	HR (95% CI)	*p*-Value
Female	0.74 (0.43,1.27)	0.272
Age	1.00 (0.98,1.01)	0.617
BMI	1.00 (0.92,1.08)	0.989
systolic	0.99 (0.98,1.00)	0.222
diastolic	0.99 (0.97,1.01)	0.328
Hypertension	1.16 (0.68,1.97)	0.583
Diabetes	1.97 (1.16,3.34)	0.014
hyperlipoidemia	1.31 (0.47,3.61)	0.605
Smoking	0.97 (0.58,1.62)	0.908
Stroke history	1.75 (1.03,2.99)	0.04
Antiplatelet history	1.46 (0.66,3.21)	0.349
Antiplatelet therapy		0.791
Aspirin	Reference	
DAPT	1.20 (0.69,2.09)	
Clopidogrel	1.29 (0.38,4.35)	
Statins therapy	1.61 (0.96,2.69)	0.071
Oral hypoglycemic agents	0.73 (0.23,2.32)	0.975
Insulin injection	0.98 (0.24,4.00)	0.591
NIHSS	1.03 (0.87,1.22)	0.704
Hours from onset	1.00 (0.99,1.01)	0.977
HCY	0.98 (0.96,1.01)	0.146
HDL	0.81 (0.29,2.24)	0.679
LDL	0.98 (0.7,1.35)	0.883
TG	1.05 (0.82,1.34)	0.717
TC	0.94 (0.73,1.21)	0.606
HbA1c	1.11 (0.96,1.28)	0.17
FPG	1.18 (0.92,1.26)	0.242
SHR per 0.1	1.34 (1.22,1.46)	< 0.001
SHR tertiles*		< 0.001
Lower	Reference	
Middle	1.19 (0.52,2.76)	
Higher	3.85 (1.91,7.75)	
ICAS	9.41 (4.98,17.77)	< 0.001

*Patients were categorized into 3 groups by tertiles of SHR (Q1–Q3).

In multivariate Cox regression analyses, to control for potential confounders, Model I was adjusted for age and sex, Model II was adjusted for age, sex, smoking, NIHSS, stroke history, antiplatelet therapy, and Model III was adjusted for age, sex, systolic pressure, diastolic pressure, BMI, NIHSS, history of stroke, hyperlipoidemia, antiplatelet medication at admission and statin therapy. Confounders were determined based on univariate analyses and previous literature reports. We first verified the effects of different glucose metrics (FPG, HbA1c, SHR and diabetes mellitus) and ICAS on stroke recurrence, as shown in **Table 4**. Higher SHR, ICAS and the presence of diabetes mellitus were all associated with stroke recurrence (HR, 5.1, 95% CI, 2.32-11.18, P<0.001; HR, 10.7, 95% CI, 5.11-22.39, P<0.001; HR, 1.97, 95% CI, 1.09-3.57, P=0.024; respectively). To further determine the combined effect of ICAS and SHR, an interaction analysis was conducted, as shown in **Table 5**. In the ICAS group, a higher SHR was an independent risk factor for stroke recurrence (HR, 8.52; 95% CI, 3.16-22.96, P<0.001). However, the effect was not observed in the non-ICAS group. We further treated SHR as a continuous variable, and each 0.1-unit increase in SHR in the ICAS group was associated with a 1.63-fold increase in stroke recurrence (HR, 1.63, 95% CI, 1.39-1.9, P<0.001). A significant interactive effect of SHR levels with or without ICAS (P for interaction was 0.002) was noted. FPG was also associated with recurrence only in the ICAS group (HR, 1.17, 95% CI, 1.08-1.26, P<0.001). In the missing data analysis, there were 4.9%, 3.4%, 3.1%, 3.1%, 3.0%, 0.33%, 0.33% missing for HCY, TC, HDL, LDL, TG, systolic pressure, diastolic pressure respectively. To avoid differences in statistical test efficacy from direct exclusion of missing values, multiple imputation was used to treat the missing values, and the results of the subsequent analysis before and after processing were consistent.

**Table 4:** Multivariate Cox regression analysis of glucose metrics and ICAS with stroke recurrence.

Variable	crude.HR 95CI	*p*-Value	Model I HR 95CI*	*p*-Value	Model II HR 95CI**	*p*-Value	Model III HR 95CI***	*p*-Value
SHR tertiles*								
Lower	1(Ref)		1(Ref)		1(Ref)		1(Ref)	
Middle	1.19 (0.52~2.76)	0.681	1.19 (0.51~2.75)	0.692	1.08 (0.71~1.98)	0.792	0.98 (0.37~2.57)	0.964
Higher	3.85 (1.91~7.75)	<0.001	3.81 (1.89~7.68)	<0.001	4.01 (1.98~8.79)	<0.001	5.1 (2.32~11.18)	<0.001
SHR per 0.1	1.34 (1.22~1.46)	<0.001	1.34 (1.22~1.46)	<0.001	1.35 (1.22~1.47)	<0.001	1.37 (1.23~1.52)	<0.001
FPG	1.18 (0.92,1.26)	0.242	1.17 (0.93,1.26)	0.244	1.09 (0.87,1.15)	0.354	1.14 (0.89~1.27)	0.258
HbA1c	1.11 (0.96~1.28)	0.17	1.1 (0.95~1.27)	0.195	1.0(0.99~1.25)	0.243	1.1 (0.93~1.29)	0.271
Diabetes Mellitus	1.97 (1.16~3.34)	0.011	1.98 (1.16~3.37)	0.013	1.98 (1.17~3.44)	0.020	1.97 (1.09~3.57)	0.024
ICAS	9.41 (4.98~17.77)	<0.001	9.32 (4.94~17.6)	<0.001	9.69 (5.46~19.2)	<0.001	10.7 (5.11~22.39)	<0.001

*Model I adjusted for age and sex; **Model II adjusted for age, sex, smoking, NIHSS, stroke history, antiplatelet therapy; ***Model III adjusted for age, sex, systolic pressure, diastolic pressure, smoking, BMI, NIHSS, Stroke history, hyperlipoidemia, antiplatelet therapy, statins therapy.

**Table 5:** Interaction analysis between glucose metrics and ICAS for stroke recurrence.

Variables	Subgroup	n.total	n.event(%)	crude.HR 95CI	*p*-Value	Model I HR 95CI*	*p*-Value	Model II HR 95CI**	*p*-Value	Model III HR 95CI***	*p*-Value	*p* forinteraction
SHR (categories)	No ICAS											0.2
	Low SHR	137	3 (2.2)	1(Ref)		1(Ref)		1(Ref)		1(Ref)		
	Middle SHR	143	5 (3.5)	1.6 (0.38~6.7)	0.519	1.63 (0.39~6.88)	0.506	1.04 (0.23~4.92)	0.501	0.51 (0.07~3.54)	0.491	
	High SHR	141	4 (2.8)	1.31 (0.29~5.86)	0.723	1.32 (0.29~5.92)	0.716	1.20 (0.24~5.93)	0.692	1.15 (0.22~5.92)	0.679	
	ICAS											
	Low SHR	66	7 (10.6)	1(Ref)		1(Ref)		1(Ref)		1(Ref)		
	Middle SHR	60	7 (11.7)	1.07 (0.37~3.04)	0.902	1.06 (0.37~3.03)	0.91	1.09 (0.56~3.23)	0.941	1.11 (0.35~3.51)	0.961	
	High SHR	63	32 (50.8)	6.24 (2.75~14.18)	<0.001	6.25 (2.74~14.26)	<0.001	7.13 (3.04~17.23)	<0.001	8.52 (3.16~22.96)	<0.001	
SHR per 0.1 (continuous)	No ICAS	421	12 (2.9)	0.96 (0.73~1.26)	0.759	0.96 (0.72~1.27)	0.76	0.97 (0.71~1.34)	0.80	0.99 (0.7~1.4)	0.945	0.002
	ICAS	189	46 (24.3)	1.45 (1.31~1.6)	<0.001	1.46 (1.32~1.62)	<0.001	1.50 (1.36~1.72)	<0.001	1.63 (1.39~1.9)	<0.001	
FPG	No ICAS	421	12 (2.9)	0.98 (0.76~1.25)	0.844	0.98 (0.76~1.25)	0.845	0.92 (0.65~1.27)	0.657	0.87 (0.59~1.29)	0.498	0.17
	ICAS	189	46 (24.3)	1.17 (1.1~1.24)	<0.001	1.18 (1.1~1.26)	<0.001	1.18 (1.05~1.25)	<0.001	1.17 (1.08~1.26)	<0.001	
HbA1c	No ICAS	421	12 (2.9)	0.99 (0.68~1.45)	0.973	0.99 (0.68~1.46)	0.97	0.89 (0.57~1.56)	0.782	0.83 (0.46~1.5)	0.544	0.49
	ICAS	189	46 (24.3)	1.07 (0.91~1.25)	0.412	1.06 (0.91~1.25)	0.447	1.06 (0.91~1.26)	0.40	1.07 (0.9~1.29)	0.44	

*Model I adjusted for age and sex; **Model II adjusted for age, sex, smoking, NIHSS, stroke history, antiplatelet therapy; ***Model III adjusted for age, sex, systolic pressure, diastolic pressure, smoking, BMI, NIHSS, Stroke history, hyperlipoidemia, antiplatelet therapy, statins therapy.

The power simulation result is shown in **Figure 1S** and **Table 4S**. When the HR is set as 1.6, a sample of 600 reaches a statistical power of greater than 0.8. This result indicates that a sample of 610 in the analysis reaches sufficient power and has the ability to detect the significant regression effect.

### 3.3 Sensitivity analysis

**Figure 2:**
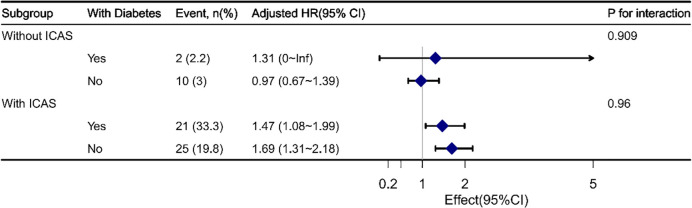
Interaction analyses between SHR and diabetes mellitus in ICAS and non-ICAS groups. No significant interaction was found between diabetes mellitus and SHR.

Sensitivity analyses were performed to clarify the impact of previous diabetes mellitus and hypoglycemic treatment, including insulin injection and oral agents (**Tables 1S**, **2S**). After further adjusting for diabetes mellitus (Model IV), each 0.1-unit increase in SHR in the ICAS group was associated with a 1.61-fold increase in the risk of an outcome event (HR, 1.61, 95% CI, 1.37-1.89, P<0.001). The significant interactive effect of SHR levels with ICAS on the primary outcome remained (P for interaction 0.002) (**Table 1S**). When further adjusting for hypoglycemic treatment (Model V), the results obtained were similar to those of Model II, III and IV (**Table 2S**). We then performed a subgroup analysis regarding whether the presence of diabetes interacts with SHR in patients with or without ICAS. No significant interaction was noted between diabetes mellitus and SHR (**Figure 2**).

**Figure 3:**
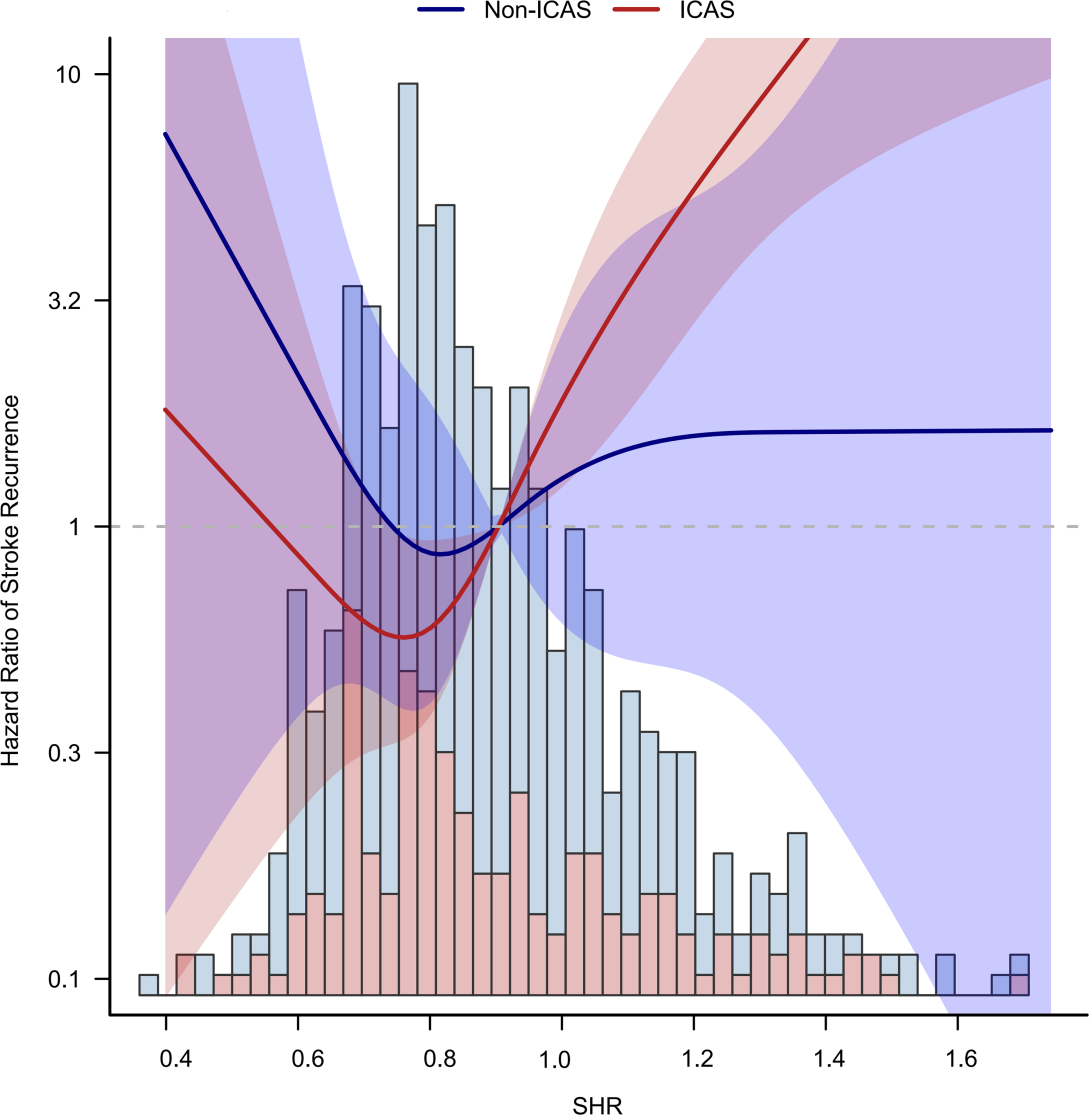
Non-liner relationship between SHR and stroke recurrence in ICAS and non-ICAS groups. A higher level of SHR over 0.75 was associated with an increased risk of stroke in the ICAS patients.

Using a Cox regression model with restricted cubic spline, we found that a higher level of SHR was associated with an increased risk of stroke with a threshold of 0.75 in the ICAS population (**Figure 3**; **Table 3S**). The association was not statistically significant in the population without ICAS.

**Figure 4:**
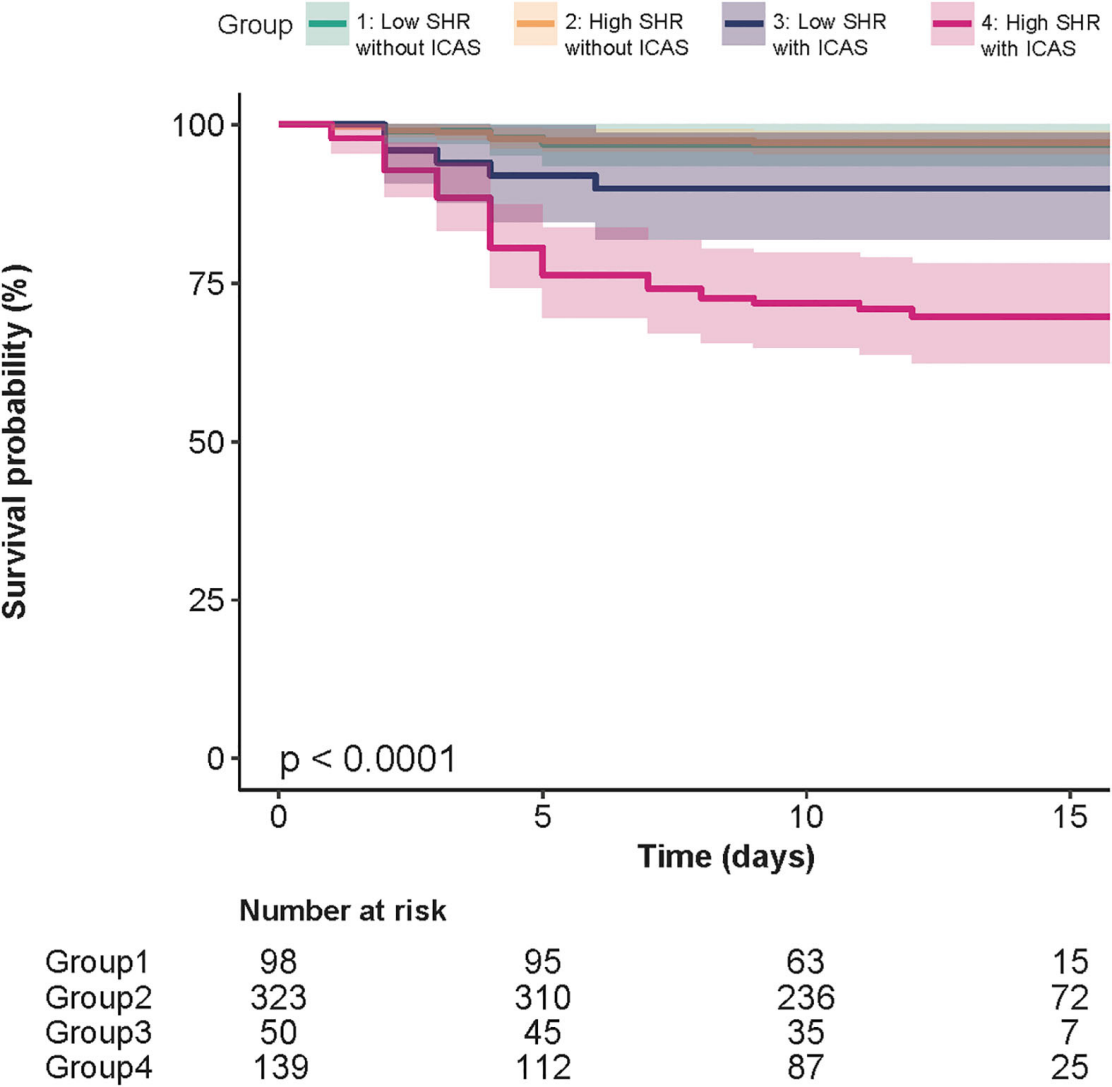
Kaplan−Meier curves of groups divided by the presence of ICAS and SHR threshold of 0.75. For patients with ICAS, recurrence mainly occurred in the first 5 days. SHR levels had minimal influence on recurrence among patients without ICAS.

Patients were divided into four groups based on the SHR threshold and ICAS status: low SHR level (<0.75) without ICAS, high SHR level (≥0.75) without ICAS, low SHR level with ICAS, and high SHR level with ICAS. We used low SHR without ICAS as a control group and performed a Cox regression analysis of the combined grouping of ICAS and SHR on the risk of stroke recurrence. The risk of stroke recurrence was increased 3.67-fold for low SHR with ICAS (HR, 3.67, 95% CI, 0.86-15.67, P=0.079). The risk of stroke recurrence was increased 9.89-fold for high SHR with ICAS (HR, 9.89, 95% CI, 2.96-33.92, P<0.001). The survival curve illustrated that for patients with ICAS, recurrence mainly occurred in the first 5 days. SHR levels had minimal influence on recurrence among patients without ICAS (**Figure 4**).

In order to determine whether the different time of onset will affect the conclusions of the study, we performed a sensitivity analysis according to the time of onset (within 24 hours of onset and within 24-72 hours of onset), and found that the time of onset did not affect the previous results (**Table 5S**).

## 4 Discussion

The results suggest that the increase in stress hyperglycemia and the presence of ICAS have a combined effect on increasing the recurrence risk during hospitalization for ischemic stroke patients.

FPG has long been considered to be a risk factor for poor prognosis of stroke. The results of our study also confirmed this finding. Traditionally, stress hyperglycemia is defined as elevated FPG in the acute phase of severe diseases, especially for nondiabetic patients, and it represents acute inflammatory and neurohormonal dysregulation, which is strongly associated with adverse outcomes and poor prognosis (8). Many studies have shown that stress hyperglycemia is independently associated with mortality and worse functional outcomes following stroke (1, 17, 18, 24). However, it is not appropriate to use elevated FPG as a biomarker of stress hyperglycemia because diabetic patients with chronic poor glucose control may be incorrectly considered to have stress hyperglycemia without taking into account background or baseline glycemia levels (10). Moreover, the presence of diabetes mellitus may also influence the power of FPG to evaluate stress hyperglycemia. Stress hyperglycemia calculated based on background blood glucose levels can better reflect the poor prognosis of severe diseases. SHR, which was first proposed by Roberts (10) and defined as admission glucose divided by estimated average glucose derived from HbA1c, helps identify the risk of critical illness better than absolute hyperglycemia, and it has been introduced to cardiovascular and stroke studies. For ST-elevation myocardial infarction patients, SHR helps predict major adverse cardiac and cerebrovascular events after percutaneous coronary intervention (25). SHR is also associated with new-onset atrial fibrillation after acute myocardial infarction (26). In a retrospective cohort study including 300 hospitalized patients with ischemic stroke, SHR at admission provided the best prognostic insight into poor outcomes compared to glycemic gap and fasting glucose (16). For stroke patients who received intravenous thrombolysis, SHR also has a better predictive value for poor functional outcome than random glucose or FPG (24). However, few studies have investigated whether stress hyperglycemia could be applied to predict the recurrence risk of ischemic minor stroke. In a secondary analysis of clopidogrel with aspirin in the Acute Minor Stroke or Transient Ischemic Attack (CHANCE) trial, researchers revealed that stress hyperglycemia was associated with a 3-month risk of recurrent stroke in minor ischemic stroke patients (17). However, their study did not take into account specific pathophysiological differences in stroke types, which may have a great impact on the relationship between stress hyperglycemia and stroke recurrence. Thus, our study hypothesized that the adverse effect of stress hyperglycemia may be more prominent in stroke patients with ICAS. The results of our study were consistent with previous reports. Our study further added evidence that stress hyperglycemia was only associated with stroke recurrence in patients with ICAS.

Our results also suggested that the SHR could estimate the risk of stroke recurrence in ICAS patients regardless of previously diagnosed diabetes mellitus. This finding is consistent with a secondary analysis of the CHANCE trial, which proved that stress hyperglycemia may have a higher risk of stroke recurrence compared to previously diagnosed diabetes mellitus or nondiabetes mellitus groups (18). However, another study conducted in ischemic stroke patients showed that the value of the SHR to predict recurrence was only significant in patients without preexisting diabetes mellitus (27). This finding seems to be inconsistent with our results. Stress hyperglycemia was traditionally defined as absolute hyperglycemia without evidence of previously diagnosed diabetes, but the limitation of the diagnostic criterion is that it did not take into account the background blood glucose level and cannot distinguish between stress hyperglycemia and diabetes mellitus. Thus, more attention should be given to the immediate severity of stress hyperglycemia rather than previously diagnosed diabetes. Another reason why the study conclusions are controversial is the different recruiting criteria among the studies. ICAS, which reflects the abnormal structure of large blood vessels, may be more sensitive to glucose stress levels.

Large artery atherosclerotic stroke (LAA) is the most common stroke subtype in the TOAST classification. In this condition, plaque rupture caused by atherosclerosis leads to platelet aggregation, which leads to thrombosis and finally to the clinical manifestation of vascular blockage (28). The CICAS study in China reported that severe stenosis and multiple stenoses represent risk factors for recurrent stroke. The risk of recurrent stroke was significantly higher in patients with ICAS (29). Mu et al. (30) found that arterial stenosis and systolic/diastolic dysfunction in the early stage of diabetes mellitus can lead to worse prognosis after ischemic stroke. We hypothesize that ICAS can be used as an indirect factor of stress hyperglycemia in stroke patients with poor prognosis. In our study, we found that the existence of ICAS is a prerequisite for stress hyperglycemia to influence stroke recurrence. This finding may be explained by the adverse consequences caused by stress hyperglycemia exaggerating the process of thrombosis after atherosclerotic rupture.

Hyperglycemia can aggravate the enlargement and rupture of atherosclerotic plaques in intracranial arteries, coronary arteries and peripheral arteries by affecting the function of brain endothelial cells, which is more likely to lead to thrombosis (31). The acute fluctuation of blood glucose levels is related to an increase in plaque instability and infarction area (32). Flynn et al. (33) found that an abnormal instantaneous increase in circulating glucose concentration promotes bone marrow formation, leads to an increase in circulating monocytes and accelerates the occurrence of atherosclerosis. Yang et al. (34) noted that the increase in the stress hyperglycemia plays an important role in predicting poor prognosis of patients with coronary heart disease undergoing percutaneous coronary intervention. For angina patients diagnosed as ischemia with nonobstructive coronary arteries, SHR at admission can significantly increase the risk of rehospitalization for chest pain (35). Given that the essence of coronary heart disease is atherosclerosis, it further suggests that the increase in the stress hyperglycemia may be involved in the rupture of atherosclerotic plaques and lead to hospital reinfarction for stroke patients. Interestingly, Dong et al. found that after middle cerebral artery occlusion, cerebral ischemia−reperfusion injury is worsened in hyperglycemic rats. Inhibition of microglial cells by stress hyperglycemia might partly explain poor outcomes after ischemic stroke (36).

This article suggested using simple calculations to evaluate the level of stress hyperglycemia for minor stroke at admission and combining imaging indicators to help further identify the risk of recurrence (23). For patients with higher SHR and severe intracranial atherosclerosis, more aggressive antithrombotic strategies, lipid-lowering treatment to stabilize the atherosclerotic plaque and intensive glucose monitoring may be needed (37).

In future research, we can further quantify the degree of intracranial artery stenosis to conduct more accurate and individualized research to help us better understand the relationship between stress hyperglycemia and intracranial artery stenosis. Furthermore, we can carry out more glucose-lowering strategies and antithrombotic strategies for this group of people to reduce the in-hospital recurrence rate of minor stroke patients with severe intracranial artery stenosis and stress hyperglycemia.

Some limitations of our study should be noted. First, as a design limitation, the results are not defined by time but vary according to the length of hospital stay. Thus, different follow-ups may bias the research results to determine the events of patients with longer lengths of stay rather than patients with shorter lengths of stay. Second, our study did not clearly define the artery responsible for the infarction but simply divided the degree of artery stenosis into less than 50% and greater than 50%. It is necessary and feasible to determine the relationship between the artery responsible for stenosis and reinfarction. We will quantify this index in future research. Third, compared with digital subtraction angiography, MRA limits spatial resolution and may increase the insensitivity to accurate assessment of arterial stenosis. Fourth, this study only included 610 patients from three provincial capital hospitals, and limited statistical ability was inevitable. Due to the high prevalence of ICAS in Asians, the universality of subgroup analysis needs to be further verified. With this in mind, research on other races is necessary to present the scalability of results on a global scale. Fifth, the impact of stress hyperglycemia on the rheological and hemodynamic characteristics of ICAS requires further investigation (4). Stress hyperglycemia may further influence key biochemical factors, such as the endothelial function and filtration rate of low-density lipoprotein, which deserve further exploration (38). By integrating the SHR into the existing stroke risk stratification tools derived from cerebral hemodynamic parameters, the effectiveness of predicting stroke recurrence will be improved (39).

## 5 Conclusion

SHR represents a better biomarker to predict the risk of stroke recurrence in patients with ICAS than FPG and HbA1c regardless of previous diabetes mellitus.

## Data availability statement

The raw data supporting the conclusions of this article will be made available by the authors, without undue reservation.

## Ethics statement

The studies involving human participants were reviewed and approved by The Ethics Committee of First Hospital of Shanxi Medical University. Written informed consent for participation was not required for this study in accordance with the national legislation and the institutional requirements.

## Author contributions

YW was a major contributor to the writing of the manuscript. YW and HongF performed the statistical analysis. WD, ZR, and XuL drafted the manuscript. XN designed and supervised the study. YL, KZ, HongF, JR and JL performed data collection. TL revised the manuscript. XL and XW supervised each stroke center. All authors contributed to the article and approved the submitted version.

## Supplementary material

Supplementary Figure 1 Post hoc power simulation analysis. A sample of 600 reaches a statistical power of greater than 0.8.

Supplementary Figure 2 Distribution of missing data. A. Histogram of missing data distribution; B. Heatmap of missing data. There were 4.9%, 3.4%, 3.1%, 3.1%, 3.0%, 0.33%, 0.33% missing for HCY, TC, HDL, LDL, TG, systolic pressure, diastolic pressure respectively.
