# Supplementary material for: Elevated stress hyperglycemia and the presence of intracranial artery stenosis increase the risk of recurrent stroke
Source: Front Endocrinol (Lausanne). 2023 Jan 9;13:954916. doi: 10.3389/fendo.2022.954916 (PMC9868694; doi:10.3389/fendo.2022.954916)
Supplement: Supplementary file 1 [file Table_1.docx]

**Supplementary tables**

**Table 1S Sensitivity analysis further adjusting for diabetes mellitus (model IV): Interaction analysis between SHR and ICAS for stroke recurrence.**

| **Variables** | **Subgroup** | **n.total** | **n.event(%)** | **Model III HR 95CI** | ***p*-Value** | ***p* for interaction** |
| --- | --- | --- | --- | --- | --- | --- |
| SHR categories | **No ICAS** |  |  |  |  | 0.2 |
|  | Low SHR | 137 | 3 (2.2) | 1(Ref) |  |  |
|  | Middle SHR | 143 | 5 (3.5) | 0.5 (0.07~3.58) | 0.491 |  |
|  | High SHR | 141 | 4 (2.8) | 1.42 (0.27~7.46) | 0.679 |  |
|  | **ICAS** |  |  |  |  |  |
|  | Low SHR | 66 | 7 (10.6) | 1(Ref) |  |  |
|  | Middle SHR | 60 | 7 (11.7) | 1.03 (0.32~3.27) | 0.961 |  |
|  | High SHR | 63 | 32 (50.8) | 7.69 (2.82~20.95) | <0.001 |  |
| SHR per 0.1(continuous) | **No ICAS** | 421 | 12 (2.9) | 1.01 (0.71~1.43) | 0.961 | 0.002 |
|  | **ICAS** | 189 | 46 (24.3) | 1.61 (1.37~1.89) | <0.001 |  |

Model IV was further adjusted for diabetes mellitus based on model III.

**Table 2S Sensitivity analysis further adjusting for hypoglycemic treatment (model V): Interaction between SHR and ICAS for stroke recurrence.**

| **Variables** | **Subgroup** | **n.total** | **n.event(%)** | **Model IV HR 95CI** | ***p*-Value** | ***p* for interaction** |
| --- | --- | --- | --- | --- | --- | --- |
| SHR categories | **No ICAS** |  |  |  |  | 0.195 |
|  | Low SHR | 137 | 3 (2.2) | 1(Ref) |  |  |
|  | Middle SHR | 143 | 5 (3.5) | 0.5 (0.07~3.46) | 0.486 |  |
|  | High SHR | 141 | 4 (2.8) | 1.44 (0.26~8.03) | 0.675 |  |
|  | **ICAS** |  |  |  |  |  |
|  | Low SHR | 66 | 7 (10.6) | 1(Ref) |  |  |
|  | Middle SHR | 60 | 7 (11.7) | 1.12 (0.35~3.61) | 0.847 |  |
|  | High SHR | 63 | 32 (50.8) | 8.57 (3.15~23.28) | <0.001 |  |
| SHR per 0.1(continuous) | **No ICAS** | 421 | 12 (2.9) | 1.02 (0.7~1.47) | 0.935 | 0.002 |
|  | **ICAS** | 189 | 46 (24.3) | 1.64 (1.4~1.92) | <0.001 |  |

Model V was further adjusted for oral hypoglycemic agents and insulin injection based on model IV.

**Table 3S SHR threshold analysis of RCS in ICAS group**

| Item | BK.HR | *p*-Value |
| --- | --- | --- |
| Estimated.Break.Point.1 | 7.5 (NA, NA) | NA |
| slope1 | 0 (0,Inf) | 0.9971 |
| slope2 | 1.798 (1.478,2.187) | < 0.001 |
| Likelihood Ratio test | - | 0.353 |
| Nonlinear Test*1 | - | 0.446 |
| Nonlinear Test*2 | - | 0.23 |

Abbreviations: RCS, restricted cubic splines

**Table 4S** **Simulated powers by sample size and effect**

|  | ia (OR.ia:1.3) | ia (OR.ia:1.6) | ia (OR.ia:1.9) |
| --- | --- | --- | --- |
| N= 200 | 0.18 | 0.449 | 0.586 |
| N= 400 | 0.331 | 0.729 | 0.907 |
| N= 600 | 0.458 | 0.884 | 0.981 |
| N= 800 | 0.576 | 0.949 | 0.996 |
| N= 1000 | 0.674 | 0.984 | 1 |

Pre-set parameters of power simulation: Prevalence of stroke recurrence among general population: 0.095; Prevalence of ICAS among general population: 0.31; Mean of SHR per 0.1 among general population: 9.1; Standard deviation of SHR per 0.1 among general population: 2.3; Main effect (HR) of ICAS: 10.7; Main effect (HR) of SHR per 0.1: 1.37; Effect (beta) of ICAS on SHR per 0.1: 0.12 ), and 1,000 simulated trials were performed.

**Table 5S Sensitivity analysis of different onset time on the relationship between SHR and ICAS for stroke recurrence.**

| **Variables** | **Subgroup** | **n.total** | **n.event(%)** | **Model II HR 95CI** | ***p*-Value** | ***p* for interaction** |
| --- | --- | --- | --- | --- | --- | --- |
| **Onset within 24 h** |  |  |  |  |  |  |
| SHR categories | **No ICAS** |  |  |  |  | 0.043 |
|  | Low SHR | 64 | 3 (4.7) | 1(Ref) |  |  |
|  | Middle SHR | 55 | 3 (5.5) | 1.14 (0.23~5.66) | 0.486 |  |
|  | High SHR | 76 | 3 (3.9) | 0.85 (0.17~4.2) | 0.675 |  |
|  | **ICAS** |  |  |  |  |  |
|  | Low SHR | 27 | 2 (7.4) | 1(Ref) |  |  |
|  | Middle SHR | 27 | 2 (7.4) | 0.96 (0.14~6.83) | 0.847 |  |
|  | High SHR | 31 | 13 (41.9) | 6.68 (1.51~29.67) | <0.001 |  |
| SHR per 0.1(continuous) | **No ICAS** | 195 | 9 (4.6) | 0.94 (0.7~1.28) | 0.935 | 0.009 |
|  | **ICAS** | 85 | 17 (20) | 1.44 (1.23~1.69) | <0.001 |  |
| **Onset within 24~72 h** |  |  |  |  |  |  |
| SHR categories | **No ICAS** |  |  |  |  | 0.076 |
|  | Low SHR | 73 | 0 (0) | 1(Ref) |  |  |
|  | Middle SHR | 88 | 2 (2.3) | 407399336.1 (0~Inf) | 0.486 |  |
|  | High SHR | 65 | 1 (1.5) | 278010891.57 (0~Inf) | 0.675 |  |
|  | **ICAS** |  |  |  |  |  |
|  | Low SHR | 39 | 5 (12.8) | 1(Ref) |  |  |
|  | Middle SHR | 33 | 5 (15.2) | 1.15 (0.33~3.96) | 0.847 |  |
|  | High SHR | 32 | 19 (59.4) | 6.27 (2.34~16.84) | <0.001 |  |
| SHR per 0.1(continuous) | **No ICAS** | 226 | 3 (1.3) | 0.99 (0.56~1.76) | 0.935 | 0.056 |
|  | **ICAS** | 104 | 29 (27.9) | 1.44 (1.27~1.64) | <0.001 |  |

**Table 6S Proportion of missing data.**

| Variable | Missing frequency | Missing percentage, % |
| --- | --- | --- |
| Systolic pressure | 2 | 0.3279 |
| Diastolic pressure | 2 | 0.3279 |
| HCY | 30 | 4.918 |
| HDL | 19 | 3.1148 |
| LDL | 19 | 3.1148 |
| TC | 21 | 3.4426 |
| TG | 18 | 2.9508 |
