# Supplementary figures and images for: Elevated stress hyperglycemia and the presence of intracranial artery stenosis increase the risk of recurrent stroke
Source: Front Endocrinol (Lausanne). 2023 Jan 9;13:954916. doi: 10.3389/fendo.2022.954916 (PMC9868694; doi:10.3389/fendo.2022.954916)

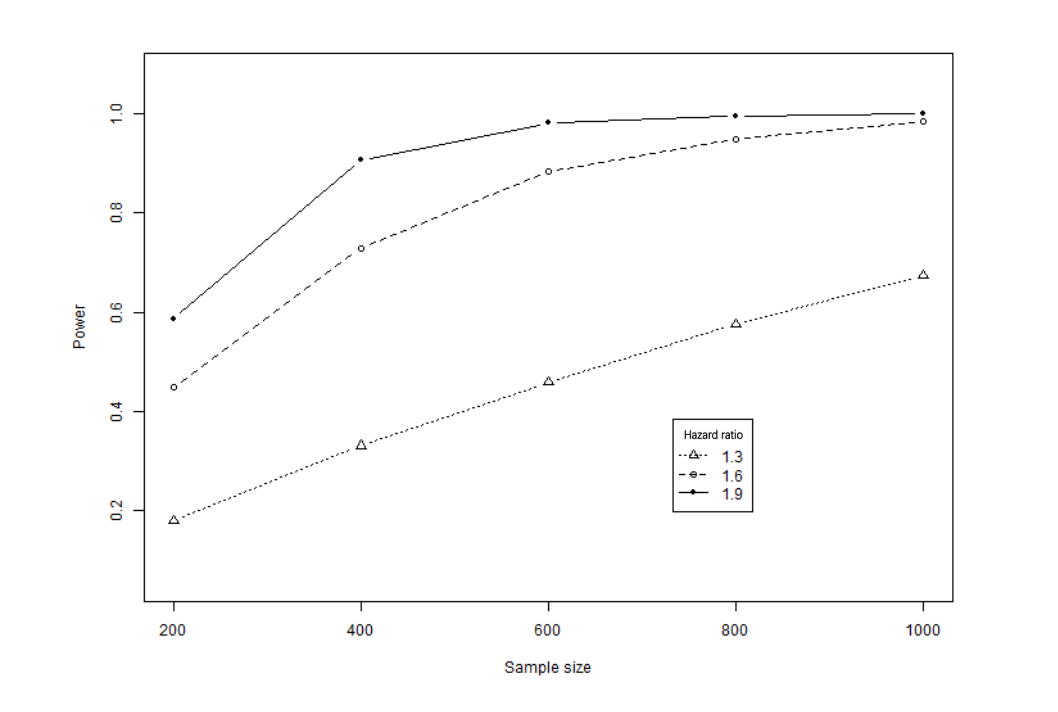

Supplement: Supplementary Figure 1 — Post hoc power simulation analysis. A sample of 600 reaches a statistical power of greater than 0.8. [file Image_1.tiff]

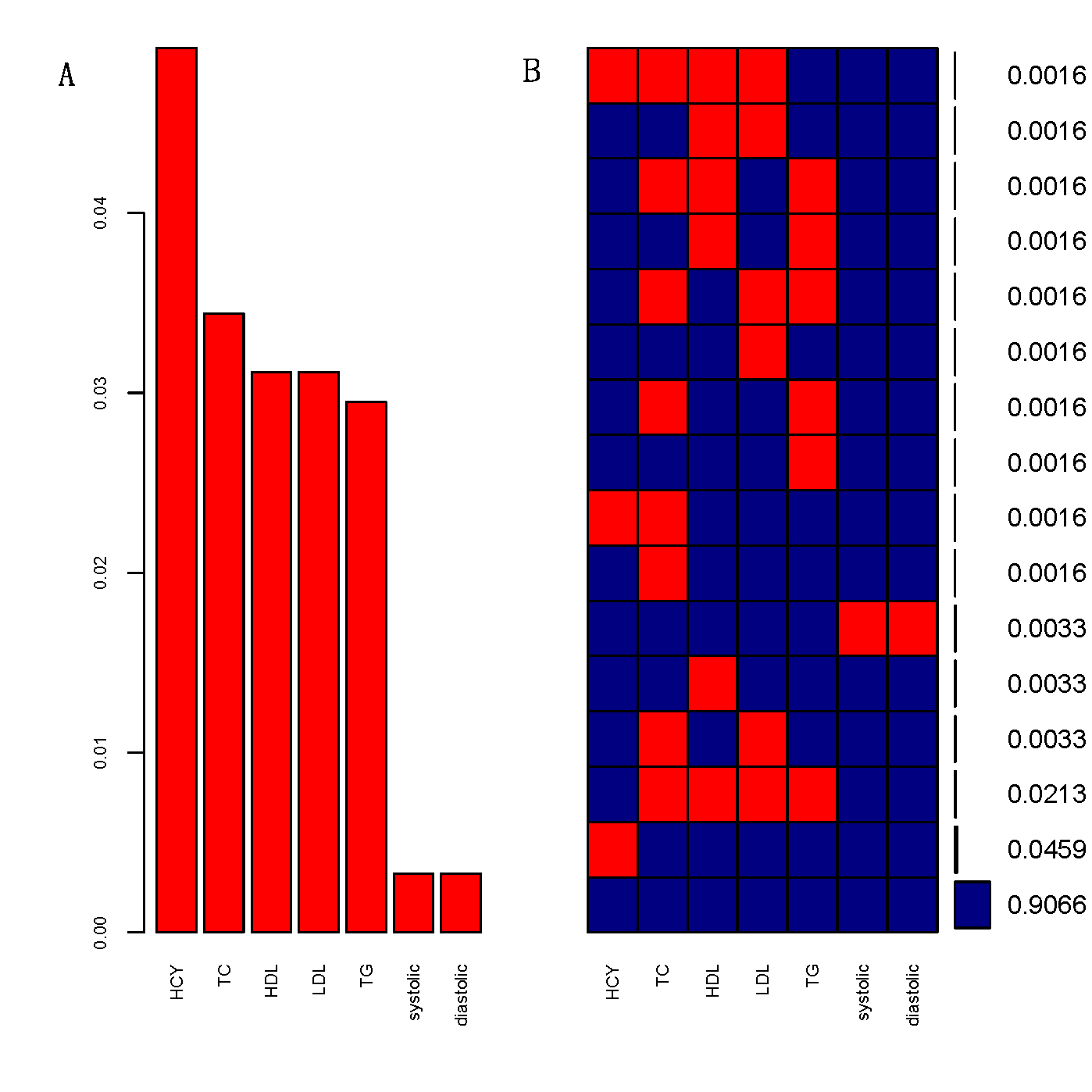

Supplement: Supplementary Figure 2 — Distribution of missing data. A. Histogram of missing data distribution; B. Heatmap of missing data. There were 4.9%, 3.4%, 3.1%, 3.1%, 3.0%, 0.33%, 0.33% missing for HCY, TC, HDL, LDL, TG, systolic pressure, diastolic pressure respectively. [file Image_2.tiff]
